# Hypertensive and cognitive function: did we come to a dead end?

**DOI:** 10.1038/s41440-022-00983-4

**Published:** 2022-07-22

**Authors:** Marijana Tadic, Cesare Cuspidi

**Affiliations:** 1 Klinik für Innere Medizin II, Universitätsklinikum Ulm, Albert-Einstein Allee 23, 89081 Ulm, Germany; 2 Department of Medicine and Surgery, University of Milano-Bicocca, Milano, Italy

**Keywords:** Hypertension, Cognitive function, late-life

The relationship between arterial hypertension and a decline in cognitive function has been investigated for decades with very conflicting results. The main question about this relationship has not yet been answered. The available data are incomparable, as populations included in these studies are heterogeneous in regard to demographic characteristics, blood pressure (BP) levels, and antihypertensive treatment (the latter is neglected in many circumstances); furthermore, concomitant diseases are often not considered, and tests used for the evaluation of cognitive function in published studies are not the same. These are the reasons for the controversial findings despite an obvious effort to answer this question.

In the current issue of the Journal, Moll et al. provided the results from the Wisconsin Longitudinal Study that followed 4314 participants who graduated high school from 1957 to 2011 [1]. The authors evaluated cognitive, demographic, and health data from 2003 to 2005 and 2011 and reported that self-reported hypertension was related to minimal to no cognitive effects in older adults. Adjustment for cardiovascular risk factors removed all associations between self-reported hypertension and cognition in the investigated population [1]. The authors separated patients with diabetes and hypertension and reported that both groups of patients did not show deterioration during follow-up (2003–2005 vs. 2011), but cognitive function slightly improved in participants without these conditions (diabetes and hypertension) [1]. The relationship between hypertension duration and cognitive performance and decline was not demonstrated in this study.

There are several important points that should be addressed to fully understand the reported findings. One of the most relevant topics is the assessment of cognitive function in the observed population. There is a lack of a standard score for this kind of evaluation in patients with hypertension or diabetes. Most cardiologists are familiar with the Mini-Mental State Test, but it seems that it cannot show small differences in cognitive function, as some other tests can [2]. The investigators in the Wisconsin Longitudinal Study evaluated cognitive function using Letter and Category Fluency, Digit Ordering, Similarities, and Immediate and Delayed Recall, which is not used often in studies that research cognitive dysfunction in hypertensive patients. Some other studies used similar scores but obtained different results [3]. In the ELSA, investigators reported that both younger and older age of hypertension, but not duration of hypertension, were related to cognitive decline in different abilities, even though participants were significantly younger (58.9 ± 5.9 years) [3]. However, the ELSA study included a multiethnic population, and only approximately half of participants were white, which is not the case with the Wisconsin Longitudinal Study, which included only a white population [1].

The large Women’s Health Initiative Memory study that included 7207 women aged 65–79 years who were followed for at least 10 years used the Telephone Interview for Cognitive Status-modified and other tests of memory, language, executive function, and working memory and reported a significant relationship between hypertension and elevated systolic (BP) and pulsed pressure and risk of mild cognitive impairment [4]. Elevated BP was significantly related to an elevated risk of mild cognitive impairment and cognitive loss, but hypertensive patients with strictly controlled BP (SBP < 120 mmHg) did not have a significantly increased risk of mild cognitive impairment (HR 1.33, 95% CI: 0.98–1.82, *p* = 0.071) or cognitive loss (1.09, (95% CI: 0.82–1.44, *p* = 0.57) in comparison with normotensive individuals [4].

The cumulative data from the ELSA and Health and Retirement Study that used the same tests for the evaluation of cognitive function included almost 17,000 participants with a median age of 62 and 65 years, respectively, during a median follow-up of 8 years and found that long-term cumulative BP was related to subsequent cognitive decline, dementia risk, and all-cause mortality in cognitively healthy adults aged >50 years [5]. The authors adjusted the models for a large number of demographic and clinical characteristics, including comorbidities and therapy.

A recently published systematic review found that hypertensive patients had worse performance in processing speed, working memory in short-term memory and learning and delayed recall [6].

The SPRINT trial provided many answers (and raised even more questions), and one post hoc analysis revealed that intensive BP control significantly reduced the risk of mild cognitive impairment and the combined rate of mild cognitive impairment or probable dementia [7]. However, there was no significant reduction in the risk of probable dementia between patients treated to a systolic BP goal of <120 mmHg and those with a goal of <140 mmHg.

The authors of the current study claimed that while the relationship between mid-life hypertension and late-life cognitive decline has been consistently demonstrated, associations between late-life hypertension and cognitive performance have been less consistent, and clarifying this was the main aim of their study [1]. However, the mean age of patients in other studies is very similar to that in this study, and investigators showed a relationship between hypertension and cognitive decline [2–4].

There are several potential reasons for this. First, self-reported hypertension is not an accurate method for the assessment of hypertension. Second, the lack of BP values in the current study does not allow for an analysis of the relationship between BP levels and cognitive decline. It is not the same if a patient has borderline or mild or severe hypertension. Third, lack of information about therapy and success of this therapy is a major limitation, as it is possible that hypertensive patients in this study were well controlled with normal BP values, and this could explain the lack of association of hypertension with cognitive impairment in this setting. Finally, the investigators also reported that the association between hypertension and cognitive decline vanished after adjustment for hypertension duration, diabetes, cardiovascular disease, hypercholesterolemia, and smoking. However, other authors included even more covariables and still demonstrated a significant relationship [2–4].

The authors of the Wisconsin Longitudinal Study separated hypertensive from diabetic patients and reported that cognitive decline was not found in these patients (either group separately) over follow-up, whereas patients without these conditions showed improvement during follow-up [1]. However, it was not clarified by the authors how cognitive function in patients without hypertension and diabetes during follow-up improved in their late life in the absence of some stimulating exercise for cognitive function. Some previous studies suggested that diabetes can be one of the main influencing factors of cognitive dysfunction in hypertensive patients [8], but this was not reported in the current investigation.

The Bayesian analytic approach used in the current study is challenging for clinicians who are used to *p* values or odds/hazard ratios. The authors claimed that this approach added further precision for detecting the obvious presence or absence of group differences and quantifying the magnitudes of the associations between hypertension and cognition. However, this also significantly complicates the understanding of their results for average clinicians who are the main readers of this kind of article.

Despite its limitations, this study showed that there are still many questions that remain to be answered in the future. This showed that the relationship between hypertension and cognitive decline is not straightforward, as we thought. However, before we even start answering these questions, we should agree about tools that will be used for evaluation of cognitive function, as it seems to be the main obstacle. Other limitations, such as establishing a diagnosis of hypertension, BP level, important clinical parameters (primarily comorbidities), concomitant therapy and other covariates, may be easily overcome with well-planned longitudinal studies.

## References

[1] Moll AC, Woodard JL. Hypertension and cognition are minimally associated in late-life. Hypertens Res. 2022. 10.1038/s41440-022-00970-9. 10.1038/s41440-022-00970-9 35787658

[2] Pan FF, Huang L, Chen KL, Zhao QH, Guo QH (2020). A comparative study on the validations of three cognitive screening tests in identifying subtle cognitive decline. BMC Neurol.

[3] de Menezes ST, Giatti L, Brant LCC, Griep RH, Schmidt MI, Duncan BB (2021). Hypertension, prehypertension, and hypertension control: association with decline in cognitive performance in the ELSA-brasil cohort. Hypertension.

[4] Liu L, Hayden KM, May NS, Haring B, Liu Z, Henderson VW (2022). Association between blood pressure levels and cognitive impairment in older women: a prospective analysis of the Women’s Health Initiative Memory Study. Lancet Healthy Longev.

[5] Li C, Zhu Y, Ma Y, Hua R, Zhong B, Xie W (2022). Association of cumulative blood pressure with cognitive decline, dementia, and mortality. J Am Coll Cardiol.

[6] Sánchez-Nieto JM, Rivera-Sánchez UD, Mendoza-Núñez VM (2021). Relationship between arterial hypertension with cognitive performance in elderly. Systematic review and meta-analysis. Brain Sci.

[7] Williamson JD, Pajewski NM, Auchus AP, Bryan RN, Chelune G, Cheung AK, SPRINT MIND Investigators for the SPRINT Research Group (2019). Effect of Intensive vs Standard Blood Pressure Control on Probable Dementia: a randomized clinical trial. JAMA.

[8] Yamamoto K, Akasaka H, Yasunobe Y, Shimizu A, Nomoto K, Nagai K (2022). Clinical characteristics of older adults with hypertension and unrecognized cognitive impairment. Hypertens Res.

